# Supplementary material for: Cost-Effectiveness of Influenza Vaccination Strategies in Adults: Older Adults Aged ≥65 Years, Adults Aged 50–64 Years, and At-Risk Adults Aged 19–64 Years
Source: Vaccines (Basel). 2022 Mar 14;10(3):445. doi: 10.3390/vaccines10030445 (PMC8955502; doi:10.3390/vaccines10030445)

Supplementary Table S1. List of ICD-10 codes used in the definition.

This study extracted broad diagnostic codes that could be used for visits to hospitals with influenza-like illness (ILI) to obtain adequate influenza incidence. ILI codes were divided into four categories: (1) acute upper respiratory disease, (2) bronchitis/bronchiolitis, (3) pneumonia, and (4) seasonal influenza.

Disease Group	Definition	ICD-10 Codes
At-risk groups	Chronic respiratory disease	J43-47, J60-65, J84
	Chronic heart disease	I05-09, I21-25, I34-37, I39.0-39.4, I42-43, I50
	Chronic renal disease	N03-08, N18-19, I12.0, I13,
	Chronic liver disease	B18, K50-51, K70.3, K71.7, K73, K74, K75.4, K76.7
	Neurologic disease	I60-69, G20-23, F00-03, G30-32, G35-37, G40-41, G70, G71, G10-14, G80-83, T91.3, R56.8
	Metabolic disease	E10-16, E24, G59.0, G63.2, N08.3, H28.0
	Autoimmune disease	M05-09, M30-36, N08.5, N16.4
	Cancer or hematologic disease	C00-97, D46, D55-64, D70-77, D80-89
	Immunosuppressed state	B20-24, D73.0
Influenza-like illness diagnostic codes	Acute upper respiratory disease	J00, J01, J02, J03, J04, J05, J06
	Bronchitis / bronchiolitis	J20, J21, J22
	Pneumonia	J12, J13, J14, J15, J16, J17.0, J17.1, J17.8, J18
	Seasonal influenza	J09, 10, 11
Acute complications related to influenza	Pneumonia	J12, J13, J14, J15, J16, J17, J18
	Encephalitis	A85.8, A86, A87.8, A87.9, A89, B94.1, G038, G039, G04.0, G04.8, G04.9, G05.1, G05.8, G36
	Myositis	M60.0, M60.1, M60.8, M60.9
	Myocarditis, pericarditis	I41.1, I51.4, I30, I31.9, I40, I51.8, B33.2
	Acute myocardial infarction	I21, I23, I24
	Stroke	I63
	Rhabdomyolysis	M62.8
	Transverse myelitis	G37.3, G37.8, G37.9
Acute exacerbation of chronic disease	Chronic respiratory disease	J41, J42, J43, J44, J45, J47
	Chronic liver disease	B18, K70.3, K71.7, K73, K74
	Chronic renal disease	N18, N19
	Chronic heart disease	I25, I42, I50
	Diabetes	E10-14

Supplementary Table S2. Daily and yearly salaries in Korea in 2018[a].

Age group	Monthly regular payment ($)[b]	Employment rate (%)	Daily regular payment ($)[b]	Yearly regular payment ($)[b]
19–24 years	1,881.72	49.5		11,177.42
25–29 years	2,461.47	78.3		23,127.97
30–34 years	3,025.09	79.5		28,859.35
35–39 years	3,470.43	78.4		32,649.81
40–44 years	3,726.70	80.0		35,776.34
45–49 years	3,830.65	82.0		37,693.55
50–54 years	3,736.56	79.9		35,826.13
55–59 years	3,368.28	75.7		30,597.45
60–64 years	2,409.50	62.6		18,100.15
19–64 years	3,165.54	49.5–82	79.36	
50–64 years	3,223.96	62.6–79.9	79.08	

[a]This table was created by reconstituting the 2018 Survey Report on Labor Conditions by employment type [1] and economically active population [2].

[b] USD 1 = KRW 1,116

Supplementary Table S3. Influenza circulation, lineage, and matching in Korea.

Season	Mismatching B [3]	Proportion of influenza type B	
		From HIMM data (unpublished)	From KDCA data [4]
2010–2011	57.1%	-	0.9%
2011–2012	27%	29.3%	48.5%
2012–2013	64.6%	9.5%	5.6%
2013–2014	85.9%	30.2%	52.9%
2014–2015	0%	25.8%	37.1%
2015–2016	97%	20.6%	51.1%
2016–2017	87.1%	-	26.6%
2017–2018	98.6%	-	54.8%
Average	64.7%	23.1%	34.7%

KDCA, Korea Disease Control and Prevention Agency; HIMM, Hospital-based Influenza Morbidity and Mortality.

Supplementary Table S4. Parameters and ranges used in sensitivity analysis.

	Base-analysis	One-way		PSA distribution
		Lower limit	Upper limit	
**Cost, $**				
**Vaccination cost of QIV (the elderly)**	28.24	22.86	37.20	Triangular
**Vaccination cost of QIV (19–64 years)**	30.95	25.22	37.20	Triangular
**Vaccination cost of ATIV**	30.55	26.70	38.25	Triangular
**Vaccination cost of HD-QIV**	30.55	26.70	38.25	Triangular
**Rapid antigen test**	17.92	10.93	46.15	Triangular
**Nursing cost (the elderly)**	51.18	−20%	+20%	Triangular
**Nursing cost (19–64 years)**	41.90	−20%	+20%	Triangular
**Transportation cost**	21.64	−20%	+20%	Triangular
**Direct medical cost**	Base	−20%	+20%	Lognormal
**Length of stay (or number of visits), days**	Base	N/A	N/A	Gamma
**Discount rate, %**	4.5	0	7.5	-
**Utilities**	Base	−20%	+20%	Triangular
**Incidence, %**	Base	−20%	+20%	Triangular

NIP, national immunization program; PSA, probabilistic sensitivity analysis.

Supplementary Table S5. Base case analysis from a societal perspective (per person cost and effectiveness) in the context of the herd effect.

		Cost (USD)	Incremental cost (ΔUSD)	Effectiveness (QALY)	Incremental effectiveness (ΔQALY)	ICER (ΔUSD/ QALY)
			**≥ 65 years**			
**At-least herd effect**	TIV	363,508,533		6,457,914		
	QIV	378,601,928	15,093,395	6,458,238	324	46,607
	ATIV	381,501,836	17,993,303	6,458,437	523	34,380
	HD-QIV	360,969,390	-2,539,143	6,458,786	873	Cost-saving
**Maximum herd effect**	TIV	349,250,952		6,458,156		
	QIV	365,845,533	16,594,581	6,458,454	298	55,628
	ATIV	369,645,487	20,394,535	6,458,639	483	42,266
	HD-QIV	350,492,321	1,241,369	6,458,964	808	1,536
			**50–64 years**			
**At-least herd effect**	Current	547,809,956		11,251,505		
	TIV	520,764,612	-27,045,344	11,252,147	641	Cost-saving
	QIV	553,742,470	5,932,514	11,252,271	766	7,762
**Maximum herd effect**	Current	524,149,925		11,251,652		
	TIV	503,398,315	-20,751,610	11,252,251	599	Cost-saving
	QIV	537,706,905	13,556,980	11,252,367	714	18,842
			**19–64 years, at-risk**			
**At-least herd effect**	Current	302,476,114		4,614,873		
	TIV	276,338,169	-26,137,944	4,615,291	418	Cost-saving
	QIV	286,330,562	-16,145,551	4,615,361	489	Cost-Saving
**Maximum herd effect**	Current	288,773,851		4,614,959		
	TIV	266,636,625	-22,137,226	4,615,350	390	Cost-saving
	QIV	277,563,848	-11,210,003	4,615,415	456	Cost-saving

1. Ministry of Employment and Labor. Employment and Labor Statistics. Available online: http://laborstat.moel.go.kr/ (accessed August 18, 2021).
2. Korean Statistical Information Service. Available online: https://kosis.kr/statHtml/statHtml.do?orgId=117&tblId=DT_11702_N083 (accessed March 28, 2021).
3. Korean Society of Infectious Disease. *Vaccinations for Adults*, 3rd ed.; Koonja Publishing: Paju, Korea, 2019, p. 184.
4. Korea Disease Control and Prevention Agency. Infectious Disease Portal. Laboratory Newsletter. Available online: http://www.kdca.go.kr/npt/biz/npp/portal/nppPblctDtaMain.do (accessed April 4, 2021)

Supplementary Figure S1. Sensitivity Analysis of Elderly Individuals A: One-way sensitivity analysis of ATIV compared with TIV. B: One-way sensitivity analysis of HD-QIV compared with TIV. C: Probabilistic sensitivity analysis of ATIV compared with TIV. D: Probabilistic sensitivity analysis of HD-QIV compared with TIV. TIV, trivalent influenza vaccine; ATIV, adjuvanted trivalent influenza vaccine; HD-QIV, high-dose quadrivalent influenza vaccine.

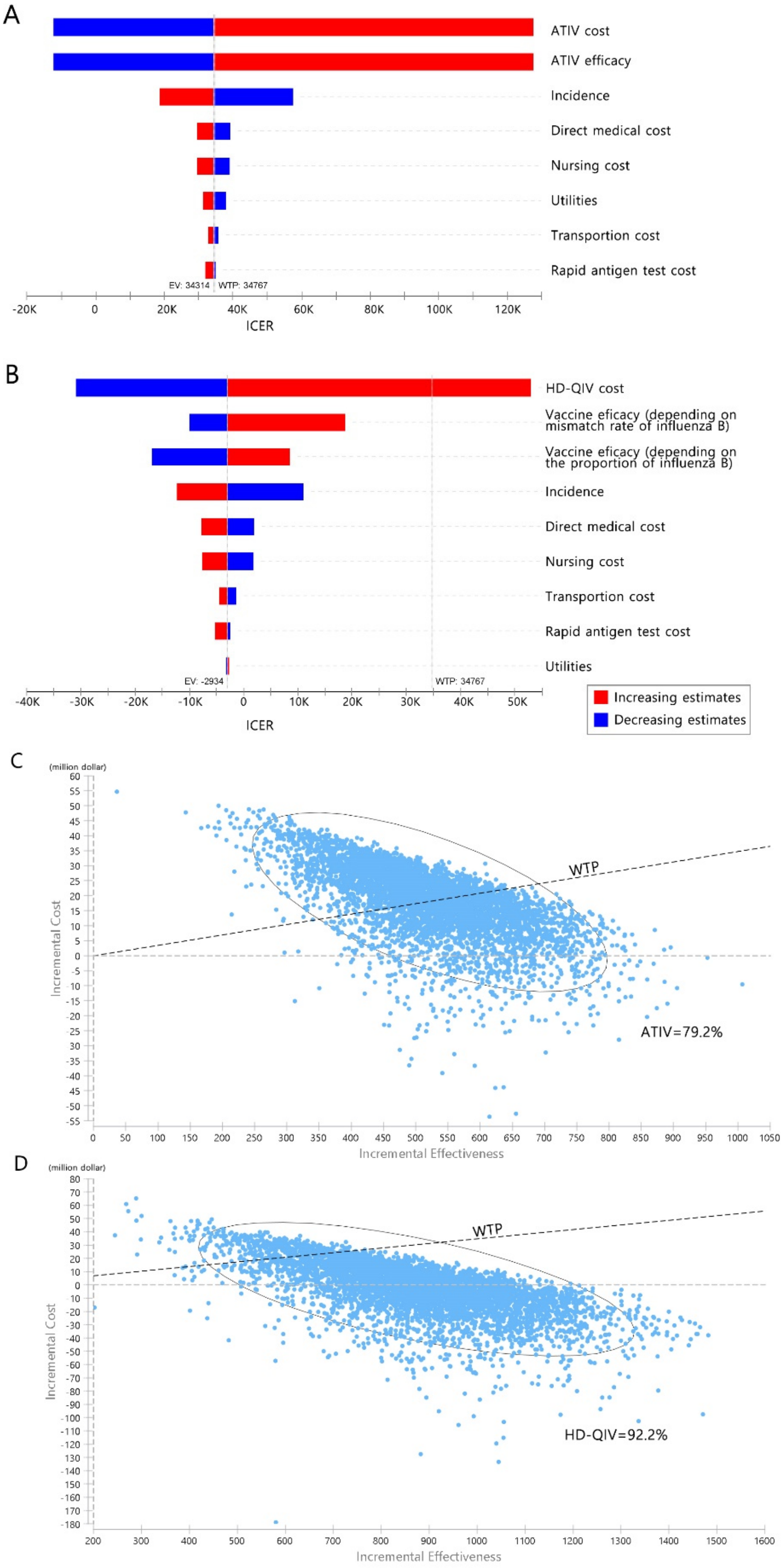

Supplement: Supplementary file 1 [file vaccines-10-00445-s001.zip › vaccines-1563556-SM.pdf]
